# MHC class II B diversity in blue tits: a preliminary study

**DOI:** 10.1002/ece3.598

**Published:** 2013-05-21

**Authors:** Juan Rivero-de Aguilar, Elske Schut, Santiago Merino, Javier Martínez, Jan Komdeur, Helena Westerdahl

**Affiliations:** 1 Departamento de Ecología Evolutiva, Museo Nacional de Ciencias Naturales (CSIC)J. Gutiérrez Abascal 2, E-28006, Madrid, Spain; 2 Behavioural Ecology and Self-Organization, The University of GroningenPO Box 11103, 9700 CC, Groningen, The Netherlands; 3 Departamento de Microbiología y Parasitología, Facultad de Farmacia, Universidad de AlcaláAlcalá de Henares, E-28871, Madrid, Spain; 4 Molecular Ecology and Evolution Lab, Ecology Building, Lund UniversitySölvegatan 37, SE-22362, Lund, Sweden

**Keywords:** *Cyanistes caeruleus*, major histocompatibility complex, *Parus caeruleus*, passerine

## Abstract

In this study, we partly characterize major histocompatibility complex (MHC) class II B in the blue tit (*Cyanistes caeruleus*). A total of 22 individuals from three different European locations: Spain, The Netherlands, and Sweden were screened for MHC allelic diversity. The MHC genes were investigated using both PCR-based methods and unamplified genomic DNA with restriction fragment length polymorphism (RFLP) and southern blots. A total of 13 different exon 2 sequences were obtained independently from DNA and/or RNA, thus confirming gene transcription and likely functionality of the genes. Nine out of 13 alleles were found in more than one country, and two alleles appeared in all countries. Positive selection was detected in the region coding for the peptide binding region (PBR). A maximum of three alleles per individual was detected by sequencing and the RFLP pattern consisted of 4–7 fragments, indicating a minimum number of 2–4 loci per individual. A phylogenetic analysis, demonstrated that the blue tit sequences are divergent compared to sequences from other passerines resembling a different MHC lineage than those possessed by most passerines studied to date.

## Introduction

The major histocompatibility complex (MHC) is a group of genes critical to immune function in vertebrates (Doherty and Zinkernagel 1975; Klein 1986). Due to its central role against infections from pathogens, the study of MHC genes has been the subject of many ecological and evolutionary studies (Sommer 2005; Spurgin and Richardson 2010), especially those studying host-parasite interactions (Westerdahl 2007; Dionne 2009). The characterization of MHC genes is constrained by its multigene nature, a result from gene duplications during evolutionary time and a turnover of new and old genes, the so-called birth-and-death model (Klein and Figueroa 1986; Nei et al. 1997; Edwards and Hedrick 1998; Nei and Rooney 2005). Thus, vertebrates have their own particularities on MHC evolution, with orthologous and paralogous genes not only maintained mainly by natural selection from parasites but also by sexual selection (Hughes and Yeager 1998). As a consequence, some MHC alleles have a long persistence time, even exceeding the species evolutionary time (i.e., trans-species polymorphism) (Klein and O'Huigin 1994).

The MHC genes constitute the most polymorphic genes among vertebrates (Piertney and Oliver 2006) and its study has been challenging, especially in non-model organisms (Babik 2010). In passerines, the study of this genetic region is also complicated, due to the existence of concerted evolution among genes (Edwards et al. 1999; Hess and Edwards 2002). Different molecular methods have been used to characterize MHC genes in birds, including PCR and non-PCR-based methods (Babik 2010). These studies involved the analysis of whole genetic region or partial regions, including complete or incomplete introns/exons. In this manner, several orders of passerine and non-passerine species have been studied to date (see Bollmer et al. 2010; Li et al. 2011; Miller et al. 2011). The first species with a completely sequenced MHC was a non-passerine, the chicken (*Gallus gallus*) and its MHC appeared simple and compact (Kaufman et al. 1999). Since then more species have been studied and the general conclusion is that the genetic organization of the passerines MHC seems to be more complex than that of non-passerines (Balakrishnan et al. 2010; Ekblom et al. 2011). This finding is supported by the derived phylogenetic position of passerines from non-passerines, where a minimal MHC seems to be the ancestral condition for birds, at least for class II genes (Hughes et al. 2008; Balakrishnan et al. 2010). Although a ratite species was observed to have at least five MHC class II loci (Miller et al. 2011). Thus, passerines have a higher number of genes, larger class I and II genes (longer introns) and also nonfunctional genes (pseudogenes) (Edwards et al. 1998; Kaufman et al. 1999; Miller and Lambert 2004a; Westerdahl et al. 2004b; Westerdahl 2007; Bollmer et al. 2010). Both MHC class I and II genes have been studied in birds involving host-parasites implications. Having an elevated/optimal number of alleles (heterozygote advantage) and/or rare advantageous alleles (negative frequency-dependent selection) would be favored by natural selection for parasite detection and elimination (Bodmer 1972; Doherty and Zinkernagel 1975; Wegner et al. 2003).

Different groups of MHC genes have been detected in different species. In the galliform birds there are two similar, but independent MHC complexes, both with class I and class II genes (Briles et al. 1993; Strand et al. 2007). The B-complex has high polymorphic and expressed genes and has been associated with disease resistance (Kaufman 2000) and the Y-complex (MHC-Y), a separate group of genes, less polymorphic, and expressed genes to a lower extent. The Y-complex has been suggested to be involved in the innate immunity (Miller et al. 2004) and controversial associations among the Y-complex and Marek's disease have been reported (Wakenell et al. 1996; Vallejo et al. 1997). In some passerines, low polymorphic genes seem reminiscent from the MHC-Y-complex (Edwards et al. 2000; Gasper et al. 2001; Bonneaud et al. 2004; Jarvi et al. 2004), therefore a phylogenetic approach has been employed to classify groups of genes (Edwards et al. 1999).

In this study, we investigate MHC class II B genes in the blue tit (*Cyanistes caeruleus*) for the first time. The blue tit is established as a model species in different ecology studies where parasite prevalence, ecological factors, and their effects on their host have been studied in depth (Hurtrez-Boussèz et al. 1997; Tripet and Richner 1997; Fargallo and Merino 1999; Merilä and Andersson 1999; Wiles et al. 2000; Merino et al. 2006; Martínez-de la Puente et al. 2010). The blue tit MHC class I genes have recently been studied (Schut et al. 2011; Wutzler et al. 2012), therefore a preliminary characterization of the MHC class II B would be a determinant for a later in-depth molecular characterization, as it gives an idea of the complexity of the system (Wittzell et al. 1994; Babik 2010). We used sequencing and restriction methods to investigate the exon 2 that codes from the most variable peptide-binding region (PBR) of the MHC class II molecule.

## Materials and Methods

### Study species

The blue tit is a small insectivorous passerine (Family Paridae) that breeds in the west Palearctic from Mediterranean to boreal zones (Cramp and Perrins 1998). Most of the geographic range (75%) of this species is located within Europe (BirdLife 2012). Blue tits willingly use nest boxes for reproduction when provided. In this study, individuals sampled came from three different European locations where individuals breed in nest boxes: Spain (Valsaín, 40°53′N, 4°01′W), Sweden (Revinge, 55°41′N, 13°26′E) and The Netherlands (The Vosbergen estate, 53°08′N, 06°35′E). The number of individuals sampled and their location of origin are detailed in Table S1.

### PCR and sequencing

#### DNA exon 2 sequences

To get a measure of MHC diversity we investigated the exon 2 that code for the β_1_ chain. The β_1_ chain encodes part of the PBR of the MHC class II molecule. To that end, whole blood samples (50–100 μL per bird) were obtained from the brachial vein and collected with a capillary tube in the field from 18 individuals (see Table S1). Blood was immediately stored in a cool box and later preserved either frozen at −80°C or in 99% ethanol, until molecular analysis. Genomic DNA was isolated either by standard phenol-chloroform extraction methods or by using the UltraClean DNA BloodSpin kit (MO BIO laboratories, Inc., CA). Genomic exon 2 sequences were amplified by polymerase chain reaction (PCR) using standard procedures with AmpliTaq DNA Polymerase PCR kit (Applied Biosystems, CA). A single PCR reaction included 25 ng of genomic DNA, 0.5 μmol/L of each of the primer 2ZFfw1 and 2ZFrv1 (Balakrishnan et al. 2010), 10× PCR buffer, 0.5 μmol/L dNTP, 2.0 mmol/L MgCl, and 1.0 unit of *Taq* polymerase in a final volume of 20 μL. The reaction was run in a thermal cycler Gene Amp PCR System 9600 (Perkin Elmer, Foster City, CA) at 94°C for 2 min, 35 cycles of (94°C for 30 sec, 60°C for 30 sec, and 72°C for 30 sec), and at 72°C for 10 min. We previously tested how general the primers were in amplifying genomic DNA from several bird species including the blue tit and also whether the primers had been utilized satisfactorily in other passerines (van Rensburg et al. 2012). The blue tit PCR product was checked on an ethidium–bromide-stained 2% agarose gel for bands of the appropriate size. Because individuals are likely to be heterozygous and the possibility of amplifying more than one allele at any locus exists, amplicons were cloned in the vector pCR2.1 with the TOPO TA Cloning kit (Invitrogen, CA) according to manufacturers' protocol. Clones with inserts were selected from colonies and diluted in 150 μL dd H_2_0 and heated to 95°C for 3 min. Up to 20 clones of each individual were used for sequencing. To do this, 1 μL of the clone dilution was amplified in a 20 μL PCR using 1 μmol/L of cloning kit primers (M13 forward and M13 reverse), 1× PCR buffer, 0.125 mmol/L dNTP, 2.0 mmol/L MgCl, and 1.0 unit of *Taq* polymerase. The amplification consisted of 35 cycles at 94°C, 60°C, and 72°C, each step for 30 sec. Positive PCR products were purified through precipitation in NH_4_Ac via centrifugation and used as template in dye terminator sequencing reactions with Big Dye Terminator (Applied Biosystems). PCR conditions consisted of 25 cycles at 96°C for 10 sec, 55°C for 5 sec, and 60°C for 4 min. After precipitation in NaAc, sequences were run on an ABI PRISM Genetic Analyzer 3730 (Applied Biosystems). Obtained sequences were read in one sequencing reaction. All sequences are the result of cloning events so each sequence corresponds to a clone. Only sequences that were found in at least two independent PCRs were regarded as verified sequences (Westerdahl et al. 2004b). Independent PCRs were performed either from the same individual or different ones. Unique sequences were also found, but were considered non verified. Non-verified sequences could not only be true sequences but also false sequences due to PCR mistakes. Primers were developed inside exon 2, therefore in this study we refer to “alleles” but they do not encompass the entire length of exon 2. In addition, we do not expect to detect all the possible alleles in the blue tit. Verified sequences were deposited in the GenBank (accession numbers JF775361 - JF775373).

#### cDNA exon 2 sequences

The purpose of this analysis was to obtain cDNA exon 2 of MHC class II B sequences from RNA and thus study transcription. Blood samples (100 μL per bird) were obtained from seven individuals again by wing vein puncture (see Table S1). Blood from individuals C, D, and E was used in both DNA and RNA analysis. Blood was preserved in 500 μL of Trizol-LS with the addition of 100 μL of K_2_EDTA following Miller and Lambert (2003). Samples were stored at 4°C for later RNA extraction. Total RNA was extracted as in Strandh et al. (2011). cDNA was obtained by RT-PCR using the two-step PCR reaction using the Retroscript kit according to protocol (Ambion, Applied Biosystems) again with the 2ZFfw1 and 2ZFrv1 primers. cDNA obtained was then used as a template on a standard PCR, where 2 μL of cDNA template was amplified with the same PCR reagents used for DNA. PCR conditions were 94°C for 2 min, 35 cycles of (94°C, 60°C, and 72°C, each step for 30 sec) and 72°C for 5 min. PCR products were cloned and sequenced as in DNA methods described above. Again verified and non-verified sequences were found. Sequences were deposited in GenBank (accession numbers JF766222 - JF766234).

### Restriction fragment length polymorphism

Another 17 individuals from two blue tit Swedish families were used for the restriction fragment length polymorphism (RFLP) analysis. These individuals were only used in this analysis and not in previous DNA or RNA analysis. With this analysis we wanted to obtain a rough description of MHC class II B genetic diversity. RFLP is based on restriction enzymes and the number of bands in each individual corresponds to the approximate number of MHC class II B alleles. To do this, we performed a restriction cleavage with PvuII on 10 μg of genomic DNA following the methods described in Westerdahl et al. (1999). The enzyme was previously tested to confirm its suitability. One of the verified cDNA sequences was used as a probe.

### Data analyses

After manual alignment in BioEdit (Hall 1999), DNA and RNA sequences were confirmed as exon 2 of MHC class II B using a BLAST search with NCBI GenBank. To measure sequence polymorphism the nucleotide diversity (π) and the number of segregating sites (S) were calculated from all alleles in DnaSP (see Librado and Rozas 2009). These values were also calculated for other passerine sequences obtained from GenBank (Table 1). The same nucleotide length was used to calculate π and S for all the species. Next a Bayesian phylogeny was performed to study evolutionary relationships among the blue tit and other passerines. For this analyses, we searched for all passerines exon 2 of MHC class II B sequences available from GenBank together with sequences corresponding to the BLB and YLB region from the chicken (*Gallus gallus*), the Eurasian black grouse (*Tetrao tetrix*), and the common mallard (*Anas platyrinchos*). From passerines functional and non-functional sequences were included. All the sequences were aligned by using MAFFT alignment implemented in Jalview (Waterhouse et al. 2009). Gblocks (Talavera and Castresana 2007) selected for the most informative nucleotide positions under the less stringent option. Redundant sequences were discarded by using the redundancy removal option in Jalview. With this option sequences with a similarity above 90% were deleted from the alignment. jModeltest 0.0.1 (Posada 2008) under corrected Akaike information criteria (AiCc) selected generalised time-reversible (GTR) as the suitable substitution model. The phylogeny was inferred in MrBayes v3.2 (Ronquist and Huelsenbeck 2003) with 80 × 10^6^ generations. The convergence of the parameter values sampled from the chains was checked by using the potential scale reduction factor (PSRF) once the standard deviation of split frequencies was below 0.01.

**Table 1:** Sequence diversity of exon 2 of MHC class II B in the blue tit and other passerines

Sp	*N*	*S*	π	SE
Agph	10	76	0.20	0.01
Pado	12	82	0.19	0.02
Apco	10	67	0.17	0.02
Anvi	13	68	0.16	0.01
Came	6	53	0.16	0.02
Pasa	3	40	0.16	0.07
Getr	15	66	0.15	0.01
Acar	7	56	0.15	0.02
Hevi	11	44	0.14	0.01
Gefo	18	57	0.13	0.01
Peau	8	45	0.12	0.01
Cyca	13	47	0.10	0.01

Sp, species; *N*, number of sequences; S, number of polymorphic sites; π, nucleotide diversity. SE, π standard deviation. Blue tit (Cyca, *Cyanistes caeruleus*), great reed warbler (Acar, *Acrocephalus arundinaceus*), red-winged blackbird (Agph, *Agelaius phoeniceus*), little greenbul (Anvi, *Andropadus virens*), Florida scrub jay (Apco, *Aphelocoma coerulescens*), house finch (Came, *Carpodacus mexicanus*), medium ground-finch (Gefo, *Geospiza fortis*), common yellow throat (Getr, *Geothlypis trichas*), Hawai'i Amakihi (Hevi, *Hemignathus virens*), house sparrow (Pado, *Passer domesticus*), savannah sparrow (Pasa, *Passerculus sandwichensis*), New Zealand robin (Peau, *Petroica australis*).

Subsequently, we also looked for signs of positive selection in the PBR. The non-synonymous (*d*_N_) and synonymous (*d*_S_) substitution ratio ω = *d*_N_/*d*_S_ provide a measure of selection pressure at the amino acid level (Nei and Kumar 2000; Yang and Nielsen 2002). Neutral genes are supposed to have a ω = 1 whereas genes under positive selection have ω > 1. We estimated ω ratio by calculating the average values of synonymous and non-synonymous substitutions per site by the Nei-Gojobori and maximum likelihood methods. In the first method, the PBR nucleotide positions were previously defined based on the MHC structure determined by crystallography (Brown et al. 1993). In this method, the Jukes Cantor correction was used (Nei and Gojobori 1986) and a *Z*-test was performed for both, codons corresponding to PBR and non-PBR. Analyses were performed in MEGA 4.0 (Kumar et al. 2008). The second method calculates the positions under selection without any *a priori* information. This analysis was performed in CODEML program included in PAML 4 package (Yang 2007). We tested the models M1a (nearly neutral), M2a (positive selection), M7 (beta) and M8 (β and ω) of codon substitutions allowing the ω ratio to vary among sites. In the analysis, a likelihood ratio test of positive selection was performed comparing model M1a against M2a and M7 against M8. *P*-values were calculated with a chi-squared test.

## Results

### MHC diversity

**Figure 1:**
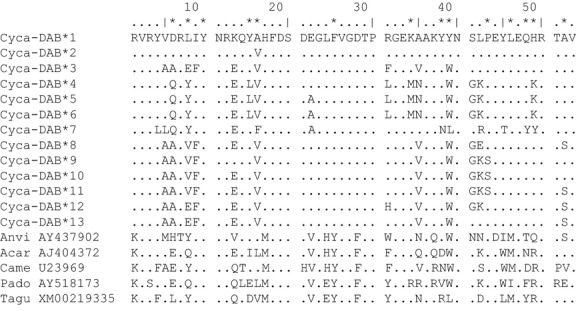
Amino acid sequences translated from blue tit exon 2 cDNA sequences and other passerines. Identity with allele Cyca-DAB*1 is indicated by *dots. Asterisks* are the codons corresponding to the PBR. Anvi, *Andropadus virens* (AY437902); Acar, *Acrocephalus arundinaceus* (AJ404372); Came, *Carpodacus mexicanus* (U23969); Pado, *Passer domesticus* (AY518173); Tagu, *Taeniopygia guttata* (XM002193356).

Overall, we obtained 217 verified sequences corresponding to blue tit exon 2 of MHC class II B from three different European locations (Table 2). A total of 96 sequences were obtained from DNA and 121 from RNA. All sequences were searched in GenBank by using BLAST and they were similar to exon 2 of MHC class II B with a maximum identity of 82%. Sequences were 159 bp long (without primers) and covered 60% of exon 2 length, which comprises 267 bp. Thirteen different alleles were verified obtained from DNA and RNA (Fig. S1). When these sequences were translated to amino acids, 12 amino acid sequences were obtained (Fig. 1). A total of 40 non-verified sequences were obtained, 38 from DNA and 2 from RNA (Table 2). No stop codon or shift in the reading frame was found in any sequence suggesting absence of pseudogenes in the samples. The maximum number of alleles found in an individual was four (individual S, Table 2), indicating the existence of at least two loci. Two individuals (Q and W) had three transcribed alleles (RNA), suggesting that both loci are expressed. Nine out of 13 alleles were found in more than one country. Alleles Cyca-DAB*3 and Cyca-DAB*9 appeared in all countries.

**Table 2:** Number of verified and non-verified exon 2 sequences found per individual

Sampling site	The Netherlands										Spain							Sweden					Total
			
Individual	A	B	C	D	E	F	G	H	I	J	K	L	M	N	O	P	Q	R	S	T	U	W	
Verified sequences																							
Cyca-DAB*1	–	–	3 (7)	–	–	–	–	–	–	–	–	–	–	–	(6)		(8)	–	–	–	–	–	3 (21)
Cyca-DAB*2	–	–	–	–	8 (11)		–	–	–	–	–	–	–	–	–	–	–	–	–	–	–	–	8 (11)
Cyca-DAB*3	–	2	–	–	–	–	–	–	–	–	–	–	–	–	(11)	–	–	3	–	–	–	–	5 (11)
Cyca-DAB*4	–	–	–	–	–	–	–	1	–	–	–	6	–	–	–	(12)	–	–	–	–	–	–	7 (12)
Cyca-DAB*5	–	–	–	–	–	–	–	–	–	–	–	–	–	–	–	–	–	2	2	–	–	(6)	4 (6)
Cyca-DAB*6	–	–	–	–	–	–	–	–	–	–	8			1	–	–	–	4	6	–	–	(5)	19 (5)
Cyca-DAB*7	–	–	–	–	–	–	–	–	1	–	–	–	–	–	–	–	–	–	1	–	1	(7)	3 (7)
Cyca-DAB*8	–	–	–	–	3 (4)	–	–	–	–	–	–	–	–	–	–	–	–	–	–	–	–	–	3 (4)
Cyca-DAB*9	–	–	–	3 (3)	–	2	4	–		4			1	–	–	–	–	–	1	1	–	–	16 (3)
Cyca-DAB*10	–	–	–	3	–	–	–	–	–	–	–	–	–	–	–	–	(3)	–	–	–	–	–	3 (3)
Cyca-DAB*11	6	–	–	5 (17)	–	–	–	–	–	–	–	–	–	–	–	(7)	–	–	–	–	–	–	11 (24)
Cyca-DAB*12	–	–	9 (7)	–	–	–	–	–	1	–	–	–	–	–	–	–	–	–	–	–	–	–	10 (7)
Cyca-DAB*13	–	–	–	–	–	–	–	–	–	–	–	–	–	–	–	–	(7)			4	–	–	4 (7)
Non-verified sequences																							
Sequence 1	–	–	–	–	–	–	–	–	–	–	–	–	19	–	–	–	–	–	–	–	–	–	19
Sequence 2	–	–	–	(2)	–	–	–	–	–	–	–	–	–	–	–	–	–	–	–	–	–	–	(2)
Sequence 3	–	–	–	–	–	2	–	–	–	–	–	–	–	–	–	–	–	–	–	–	–	–	2
Sequence 4	–	–	–	–	–	–	–	–	–	–	–	–	–	9	–	–	–	–	–	–	–	–	9
Sequence 5	–	–	–	–	–	–	–	–	–	–	–	2	–	–	–	–	–	–	–	–	–	–	2
Sequence 6	–	–	–	–	–	–	–	–	–	–	–	–	2	–	–	–	–	–	–	–	–	–	2
Sequence 7	–	–	–	–	–	–	–	–	2	–	–	–	–	–	–	–	–	–	–	–	–	–	2
Sequence 8	–	–	–	–	–	–	2	–	–	–	–	–	–	–	–	–	–	–	–	–	–	–	2

Numbers without brackets, sequences obtained from DNA. Numbers in brackets, cDNA sequences obtained from RNA. Alleles: total number of verified alleles in an individual.

**Figure 2:**
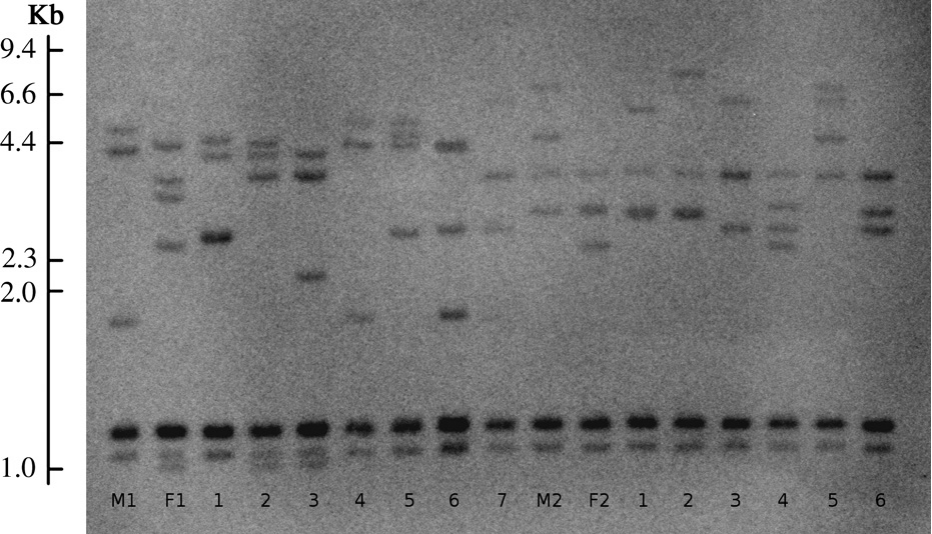
RFLP patterns with the restriction enzyme Pvu II and the blue tit MHC class II exon 2 probe, from 17 blue tit individuals corresponding to two Swedish families. M, mother; F, father; numbers are the offspring. Family 1, individuals from F1 to 6. Family 2, individuals from F2 to 13. There are 4–7 fragments per individual in the size range 1–9 kb.

The RFLP pattern consisted of 4–7 fragments per individual in the length range of 1–9 kb, each band corresponding to approximately one allele (Fig. 2). The intensity of the RFLP fragments were variable, either because their similarity to the probe differed or because RFLP fragments of certain lengths were more numerous than others (Westerdahl 2003). Nearly all individuals had unique RFLP patterns except for the individuals F1 and 4 that shared the same bands, a likely result when comparing individuals from the same family. These results suggest a minimum number of four loci. The RFLP bands may correspond to both coding and non-coding genes, so it is possible that we are observing bands corresponding to pseudogenes that we do not amplify with our primers. On the other hand, one hybridizing fragment could represent two genes with the same electrophoresis migration distance. Nucleotide diversity results from the blue tit and other passerines are presented in Table 1.

### Phylogenetic analysis of class II B sequences

**Figure 3:**
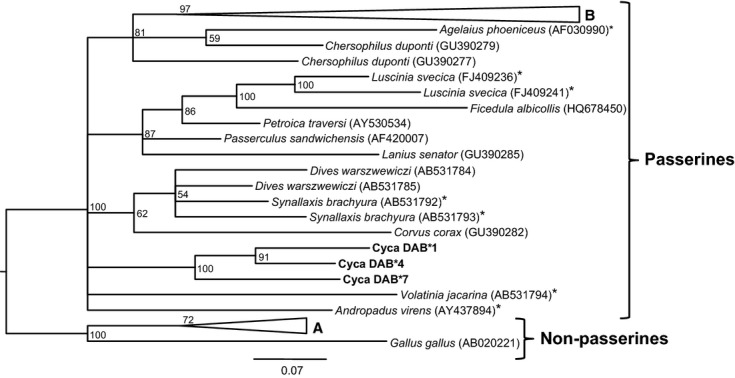
Bayesian phylogeny of exon 2 of MHC class II B sequences (based on 159 bp) from blue tit and other passerine and non-passerine species. Numbers on branches, the posterior probabilities values. *, Non-functional MHC class II. Codes following species name, GenBank accession numbers. Collapsed group A included the following: *Acrocephalus arundinaceus* (AJ404371, AJ404373, AJ404375, AJ404377, U24405, U24406, U24408), *Agelaius phoeniceus* (AF030987, AF030989, AF030994, U23970, U23971), *Andropadus virens* (AY437889, AY437890, AY437891, AY437892, AY437893, AY437900, AY437901, AY437904, AY437907, AY437908, AY437911, AY437912, AY437913), *Aphelocoma coerulescens* (U23958, U23959, U23961, U23962, U23963, U23966, U23972, U24401), *Atlapetes rufinucha* (AB531732, AB531795), *Cactospiza pallida* (AB531504, AB531518, AB531520), *Camarhynchus parvulus* (AB531822), *Carpodacus mexicanus* (AF241547, HQ203000), *Catamenia inornata* (AB531796), *Certhidea olivacea* (AB531513), *Coryphospingus cucullatus* (AB531639, AB531729, AB531741, AB531744, AB531800, AB531802), *Dendroica adelaidae* (AB531663), *Erithacus rubecula* (GU390284*), Erythrura gouldiae* (EF535335, EF535337, EF535338, EF535342, EF535348, EF535448), *Euphonia musica* (AB531807), *Ficedula albicollis* (HQ678313, HQ678323, HQ678328, HQ678338 HQ678340, HQ678492), *Ficedula hypoleuca* (GU390232, GU390233, GU390235, GU390236, GU390238, GU390241, GU390248), *Geothlypis trichas* (GQ247570, GQ247571, GQ247574, GQ247575, GQ247588, GQ247597, GQ247612, GQ247617), *Lanius senator* (GU390287), *Lonchura striata* (L42334, L42335), *Loxigilla noctis* (AB531642), *Luscinia luscinia* (FJ529849, FJ529850, FJ529851, FJ529852, FJ529854, FJ529856, FJ529857), *Luscinia svecica* (FJ529861, FJ529863, FJ529870, FJ529871, FJ529876, FJ529878, FJ529883, GQ403040), *Passer domesticus* (AY518171, AY518172, AY518173, AY518176, AY518178, AY518179, AY518180, AY518181), *Passerculus sandwichensis* (AF420008), *Petroica australis* (AY428561, AY428562, AY428563, AY428564, AY730420), *Petroica traversi* (AY258333, AY258335), *Pheucticus aureoventris* (AB531787), *Phylloscopus collybita* (GU390293), *Poephila acuticauda* (EF535365, EF535366, EF535382, EF535416, EF535472, EF535493), *Ramphocelus carbo* (AB531656, AB531742), *Sicalis flaveola* (AB531517, AB531809), *Sporophila nigricollis* (AB531631, AB531812), *Sturnella bellicosa* (AB531813), *Tiaris bicolor* (AB531814), *Tiaris obscura*,(AB531577, AB531633, AB531634, AB531698), *Volatinia jacarina* (AB531640, AB531743, AB531819, AB531820) and *Zonotrichia capensis* (AB531665). Collapsed group B included: *Coturnix japonica* (AB181862), *Gallus gallus* (U91532, AJ248572), *Meleagris gallopavo* (AJ616892, AM233872), *Numida meleagris* (DQ885563), *Pavo cristatus* (AY928098), *Tetrao tetrix* (EF174544), *Phasianus colchicus* (AJ224344, AJ224346, AJ224347, AJ224349).

The Bayesian phylogeny on passerine MHC class II B sequences is presented in Figure 3 (collapsed tree) and Figure S2 (extended tree). As expected, there were two well supported clades in the tree separating passerines from non-passerines. Inside passerines there was a well supported cluster (posterior probability of 97) with the majority of the passerine sequences (139 sequences out of 155). Inside this cluster there were well supported groups but also several sequences not resolved forming a polytomy. The sequences in this group included both non-functional alleles and alleles well assigned to be involved in peptide presentation. Interestingly, all the blue tit sequences appeared outside this major group and they formed a monophyletic cluster (posterior probability of 100) together with other passerine sequences. These sequences corresponded to the Dupont's lark (*Chersophilus duponti*) GU390279, the collared flycatcher (*Ficedula albicollis*) GU390277, woodchat shrike (*Lanius senator*) GU390285, and common raven (*Corvus corax*) GU390282, corresponding to sequences obtained with degenerated primers (Canal et al. 2010). Among these sequences non-functional sequences were intermingled as well, that is, red-winged blackbird (*Agelaius phoeniceus*) AF030990, bluethroat (*Luscinia svecica*) FJ409236, FJ409241, slaty spinetail (*Synallaxis brachyura*) AB531785, AB531793, blue-black grassquit (*Volatinia jacarina*) AB531794, and little greenbul (*Andropadus virens*) AY437894.

### Selection analysis on exon 2

Putative PBR codons were inferred from HLA and then we did ω ratio analysis for the PBR and non-PBR, respectively, to detect selection. For the PBR, non-synonymous substitutions exceeded those of synonymous substitutions ([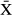 ± SE] *d*_N_ = 0.283 ± 0.093, *d*_S_ = 0.134 ± 0.062, ω = 2.11), hence there is a tendency for positive selection although this was not significantly different from 1 (*Z* = 1.566; *P* = 0.060). For the non-PBR, non-synonymous substitutions were similar to the synonymous substitutions (*d*_N_ = 0.076 ± 0.026, *d*_S_ = 0.078 ± 0.033, ω = 0.97) and not significantly different from 1 (*Z* = −0.050; *P* = 1). For all positions (PBR and non-PBR together), non-synonymous substitutions exceeded those of synonymous substitutions (*d*_N_ = 0.123 ± 0.029, *d*_S_ = 0.090 ± 0.028, ω = 1.37), also not significantly different from 1 (*Z* = 0.016, *P* = 0.181).

Maximun likelihood methods in CODEML found evidence of positive selection in the exon 2. Models involving selection (M2a and M8) fits the data significantly better than their respective neutral models (M1a and M7). Bayes empirical Bayes (BEB) found the amino acid sites 8, 31, and 42 to be under positive selection (ω > 1). These positions were selected by both models M2a and M8. Position 8 fitted with that designated by Brown et al. (1993) to be a PBR position, whereas position 42 was one amino acid next to a designed PBR position. Position 31 coincided with a non-PBR position.

## Discussion

### MHC diversity

In this study, we have examined the exon 2 of MHC class II B genes in the blue tit for the first time. We found a maximum of three expressed alleles per individual detected by sequencing and a maximum of seven alleles detected by RFLP, suggesting at least two to four loci in the blue tit. We are cautious about the results observed and we propose them conservative and preliminary, as primers were designed over zebra finch sequences MHC class II sequences and are likely not amplifying all possible alleles. We checked this possibility adding new sequences to the dataset from recently sequenced species and several sequences were not detected, therefore blue tit MHC may be underestimated. Also, the values found for nucleotide diversity should be taken with caution, as the fact that non-verified sequences were amplified adverts that nucleotide diversity could increase if these sequences become corroborated. Taken this into account, we compared our results with that found in other birds. The total number of MHC class II B loci described in other passerines ranged from three (house sparrow and the red-winged blackbird) to 20 (common yellowthroat) and the number of transcribed loci ranged from three (house sparrow and the scrub jay) to eight (common yellowthroat) (see Bollmer et al. 2010). And the nucleotide diversity for exon 2 genes ranged from 0.007 (green bull) to 0.19 (house sparrow) (Bonneaud et al. 2004; Aguilar et al. 2006). When the blue tit sequence diversity was compared with some other passerines after controlling for the sequence length, a similar polymorphism was found (see Table 1). In the blue tit we found a value of π = 0.10, characteristic of polymorphic genes, although groups of sequences with low polymorphism have been found in some passerines (Edwards et al. 2000; Gasper et al. 2001; Bonneaud et al. 2004; Jarvi et al. 2004). But the values found in these cases are so low that the lack of polymorphism is evident (i.e., the low π value found in the greenbul). Likewise, similar values of blue tit π have been found in non-passerines, that is, in the chicken (0.107) or the black grouse (0.113) (Strand et al. 2007). Thus, blue tit MHC diversity is similar to other passerines, but resembling less diverse MHC genes. A low MHC class I genetic diversity in the blue tit has been reported by Schut et al. (2011) and Wutzler et al. (2012).

Several different hypotheses could explain the MHC diverse observed in the blue tit. A reduced MHC genetic diversity could be due to the population bottleneck the blue tits suffered during the last ice age (Kvist et al. 2004). In vertebrates, a lack of MHC variability has been attributed to bottleneck events (Babik et al. 2009; Becker et al. 2009) and some bird species with low MHC diversity have been reported to have passed a population bottleneck (Richardson and Westerdahl 2003; Miller and Lambert 2004b; Zhang et al. 2006; Bollmer et al. 2007). On the other hand, bird species affected by a greater diversity of parasites, either in time or space, should be selected to maintain/develop a more diverse MHC. Geographical variation in pathogen antigens leads to differential selection by same pathogens in different areas (Jeffery and Bangham 2000), therefore, a low diversity of the MHC could be explained by local adaptation to parasites (Klein 1991; Westerdahl et al. 2005; Bonneaud et al. 2006). In this respect, we can expect that migratory birds exposed to a higher diversity of parasites (Møller and Erritzøe 1998; Westerdahl et al. 2004a) could evolve a more diverse MHC than nonmigratory birds. The blue tit is considered a resident bird in Europe, except some migratory individuals from northern locations and some altitudinal movements produced during winter (Cramp and Perrins 1998). Thus, when considering the passerine species included in this study there are some birds that fits with this prediction (migratory birds with higher diversity), but is not consistent among others. It will be necessary to increase the number of species in a comparative analysis to look for the potential effect of migratory behavior on MHC genes diversity. Finally, it will be important to identify the composition of parasite lineages present in any blue tit population and which MHC alleles are conferring resistance (Westerdahl et al. 2005; Martínez-de la Puente et al. 2011). In this way, we found some blue tit alleles shared between locations, a pattern consistent with gene flow among blue tit populations across Europe (Taberlet et al. 1992; Kvist et al. 2004).

MHC diversity is critical on antigen detection and affects host response to face pathogens, but reports in this respect are ambiguous. The heterozygote advantage predicts that having more MHC alleles increases the chance on antigen recognition and the negative frequency-dependent selection stands that specific MHC alleles are advantageous against parasite lineages adapted to common MHC alleles. Therefore, different situations are expected to be found among species. Some vertebrate species with low MHC diversity are particularly susceptible to diseases (Mainguy et al. 2007) although some species are not, as is reflected by their large population size or recent expansions (Babik et al. 2009; Radwan et al. 2010). In birds, the chicken “minimal essential” MHC genes confer strong protection against Marek's disease, but a huge mortality is produced among susceptible individuals. Some vertebrates, with reduced numbers of MHC genes showed highly divergent alleles, which might allow for the recognition of a wider range of pathogens (Sommer 2005). Also variations in the level of polymorphism have been found to differ among bird species, thus some species with a unique class II B loci had more alleles than a species with more loci (Hughes et al. 2008). Thus, the expression of a single gene could result in differences in resistance and susceptibility to infectious pathogens in individuals with different MHC haplotypes (Hepkema et al. 1993; Kaufman 2000).

### Blue tit phylogenetic analyses

MHC genes can be defined evolutionarily by clustering with respect to other known MHC genes in a phylogenetic context (Hess and Edwards 2002). However, in the phylogeny based on bird MHC class II exon 2 the blue tit sequences were not clearly related to other well characterized sequences nor to non-functional ones. Instead they appeared to form a monophyletic basal group not clearly related to any sequences or group of sequences. Well characterized class II B sequences from other passerines grouped into a separate clade (group A in Fig. 3) and not close to blue tit sequences. Inside group A, several polytomies and low supported groups of sequences were observed. Similar to our results, Balakrishnan et al. (2010) found a class II B zebra finch lineage situated at the base of other passerine class II sequences in a phylogeny performed with the same primers. They suggest that there is a novel locus that has not previously sequenced in birds, but it is not known whether it is expressed or polymorphic. The phylogeny in Balakrishnan et al. (2010) was performed concatenating together exon 2 and 3 whereas in this study only the exon 2 was used. Phylogenies based on the exon 2 are less congruent than those based on exon 3 or both exon 2 and 3 combined (Hughes and Yeager 1998; Miller et al. 2011) because exon 2-based trees may reflect selection rather than a duplication history (Reusch and Langefors 2005). In this manner, it is interesting that we found a similar basal lineage separated from the rest of passerines even though our phylogeny was based only on exon 2. In the tree none of the sequences from other passerines mixed together with the blue tit sequences, therefore, the blue tit sequences could be divergent with respect to those of other passerines and represent a different cluster of genes. This mean that closely related species have not had enough time to diverge and sequences remain similar and/or concerted evolution is acting then alleles are sometimes intermingled on trees (Vincek et al. 1997; Richardson and Westerdahl 2003; Jarvi et al. 2004). Therefore, it will be necessary to include data from species closely related to the blue tit (to date not available) in the phylogeny to confirm this possibility. Also, blue tit genes could be paralogous with respect to the other passerines included in the analyses and the dissimilarity among genes then is because they did not descend from the same ancestral gene. On the other hand, selective pressures from pathogens shared between two bird species could be counteracting genetic differences between species (Westerdahl 2007) and as a consequence they would group together in the phylogeny.

### Selection on exon 2

A method to confirm the effect of different selection pressures on the blue tit MHC is to look for signs of selection on the PBR. In the analysis in MEGA, where PBR positions where a priori assigned, we found that for the PBR d_N_ is higher than *d*_S_ (indicative of positive selection) and the difference was nearly significant (*P* = 0.06). On the other hand, the analysis in CODEML found several amino acid positions under positive selection. Thus, there seems to be some positive selection acting in the PBR of blue tit exon 2, but it is not very strong. Accordingly, we cannot reject the possibility that we are misidentifying PBR positions as they are estimated from results of a crystallographic study of human MHC class II B (Brown et al. 1993) and, therefore, they are not necessarily the same positions as in the avian MHC class II B. In this respect, in other avian studies where PBR positions were estimated following Brown et al. (1993), positive selection was detected. Also a lack of strong selection on the PBR could be due to the fact that we are including several non homologous loci in the analyses. In this study, we did not assign sequences to a locus due to the similarity among sequences, therefore, the ω ratio could be affected. In addition, we have only data of the PBR and non-PBR positions of one part of exon 2 and the estimation of the ω may vary when the entire exon is amplified. The observation of trans-species polymorphism can be used to infer positive selection because it retains alleles for a long time (Bernatchez and Landry 2003). Contrary to other species trans-species polymorphism was not observed in the blue tit. Selection has been found at different temporal scales, thus selection in the distant past can be detected as an excess of non-synonymous substitutions. However, selection in the recent past or in the current generation can be detected considering other factors (e.g., measuring deviations from Hardy–Weinberg or finding correlations between disease resistance and MHC-allele or genotype (see Wegner et al. 2003). It will be necessary to incorporate these variables to correctly assess the effect of selection in the blue tit and to rule out the possibility that the soft selection could be signalling a change from a functional to a non-functional gene (Hughes and Nei 1989).

## Conclusions

With this study we have obtained a preliminary overview of the MHC class II B genes in the blue tit, characterizing exon 2 partially from several individuals originating in three European locations. Our primers were developed based on transcript sequences obtained from zebra finch RNA and they are supposed to amplify expressed class II B genes involved in peptide presentation. Primers even designed for the zebra finch are not very restricted to this species and could be considered general to be applied in the blue tit. Using the same-species probes has revealed different diversity in MHC complexity among songbirds (Edwards et al. 1999). The combined results from sequencing and RFLP can be extremely valuable at the initial stages on MHC research in non-model vertebrates, until a new molecular method like next-generation sequencing based method could be developed (Babik 2010). New molecular tools are proving that some variability is not measured by traditional molecular methods (Oomen et al. 2013), but to date, no whole genome sequencing has been performed on this species. Although the methodology employed in this study is now increasingly being substituted by next-generation sequencing is still possible to preliminarily characterize MHC in avian species or to reveal functional variation across populations (Whittaker et al. 2012). Now, more species are being studied by applying new molecular tools and this approach promises a fascinating advance in MHC study. This is the first step toward a better understanding of the MHC class II B genes in the blue tit.

## Supporting Information

**Figure S1.** Total MHC class II B blue tit alleles obtained from all DNA and cDNA sequences (amplified with the degenerated primers 2ZFfw1 and 2ZFrv1). Identity with allele Cyca-DAB*1 is indicated by *dots*.

**Figure S2.** Bayesian phylogeny from the blue tit and other passerines with non-collapsed nodes. Decimal numbers on branches, posterior probability. MHC class II B blue tit alleles are indicated by an arrow.

**Table S1.** Sample sites, number of individuals sampled and kind of analysis. +, the individual was used for the analysis. −, the individual was not used for the analysis.
