# Supplementary material for: MHC class II B diversity in blue tits: a preliminary study
Source: Ecol Evol. 2013 May 21;3(7):1878–89. doi: 10.1002/ece3.598 (PMC3728931; doi:10.1002/ece3.598)
Supplement: Supplementary file 1 [file ece30003-1878-SD1.pdf]

Supporting information

Table 1. Sample sites, number of individuals sampled and kind of analysis. + = the individual was used for the analysis. - = the individual was not used for the analysis.

Sampling site	The Netherlands										Spain							Sweden				
Individuals	A	B	C	D	E	F	G	H	I	J	K	L	M	N	O	P	Q	R	S	T	U	W
DNA Analysis	+	+	+	+	+	+	+	+	+	+	+	+	+	+	-	-	-	+	+	+	+	-
RNA Analysis	-	-	+	+	+	-	-	-	-	-	-	-	-	-	+	+	+	-	-	-	-	+

Figure legends

Fig. 1:

Total MHC class II B blue tit alleles obtained from all DNA and cDNA sequences (amplified with the degenerated primers 2ZFfw1 and 2ZFrv1). Identity with allele Cyca-DAB*1 is indicated by *dots.*

Fig. 2:

Bayesian phylogeny from the blue tit and other passerines with non-collapsed nodes. Decimal numbers on branches = posterior probability. MHC class II B blue tit alleles are indicated by an arrow.

Fig. 1

```
                       10         20         30         40         50         60         70         80
              ....|....| ....|....| ....|....| ....|....| ....|....| ....|....| ....|....| ....|....|
Cyca DAB*1    CGGGTGCGGT ACGTGGACAG GCTCATTTAC AACCGGAAGC AGTACGCGCA CTTCGACAGC GATGAGGGGC TGTTCGTGGG
Cyca DAB*2    .......... .......... .......... .......... ......T... .......... .......... ..........
Cyca DAB*3    .......... ...C..CG.. .GAAT.C... ......G... ......T... .......... .......... ..........
Cyca DAB*4    ......A... .....C.G.. .TA....... ......G... ..CT..T... .......... .......... ..........
Cyca DAB*5    ......A... .....C.G.. .TA....... ......G... ..CT..T... .......... ....C..... ..........
Cyca DAB*6    ......A... .....C.G.. .TA....... ......G... ..CT..TA.. .......... ....C..... ..........
Cyca DAB*7    ......A... TGC..C.G.. .TA....... ......G... .....TTC.. .......... ....C..... ..........
Cyca DAB*8    .......... ...C..CG.. .G.AT.C... ......G... ......T... .......... .......... ..........
Cyca DAB*9    .......... ...C..CG.. .G.AT.C... .......... ......T... .......... .......... ..........
Cyca DAB*10   .......... ...C..CG.. AG.AT.C... ......G... ......T... .......... .......... ..........
Cyca DAB*11   .......... ...C..CG.. AG.AT.C... ......G... ......T... .......... .......... ..........
Cyca DAB*12   .......... ...C..CG.. .GAAT.C... ......G... ......T... .......... .......... ..........
Cyca DAB*13   .......... ...C..CG.. .GAAT.C... ......G... ......T... .......... .......... ..........

                       90        100        110        120        130        140        150
              ....|....| ....|....| ....|....| ....|....| ....|....| ....|....| ....|....| ....|....
Cyca DAB*1    GGACACCCCG CGTGGGGAGA AGGCGGCCAA GTACTATAAC AGCCTACCGG AATACTTGGA GCAACATAGG ACTGCGGTG
Cyca DAB*2    .......... .......... .......... .......... .......... .......... .......... .........
Cyca DAB*3    .........C TT...A.... ...T...... .....GG... .......... .......... ......C... .........
Cyca DAB*4    .......... .T........ T.AAT..... .....GG... G..AA..... .......... ....A.G... .........
Cyca DAB*5    .......... .T........ T.AAT..... .....GG... G..AA..... .......... ....A.G... .........
Cyca DAB*6    .......... .T........ T.AAT..... .....GG... G..AA..... .......... ....A.G... .........
Cyca DAB*7    .......... .......... .......... .A...TG... ....G...A. ..ACAC.... .T.CT..C.. .........
Cyca DAB*8    .......... .......... ...T...... .....GG... G..GA..... .......... ......C... ...T.....
Cyca DAB*9    .......... .......... ...T...... .....GG... G..AA.T... .......... .......... .........
Cyca DAB*10   .......... .......... ...T...... .....GG... G..AA.T... .......... .......... .........
Cyca DAB*11   .......... .......... ...T...... .....GG... G..AA.T... .......... ......C... ...T.....
Cyca DAB*12   .......... .A........ ...T...... .....GG... G..AA..... .......... .......... ...T.....
Cyca DAB*13   .......... .......... ...T...... .....GG... .......... .......... .......... ...T.....
```

Fig.2

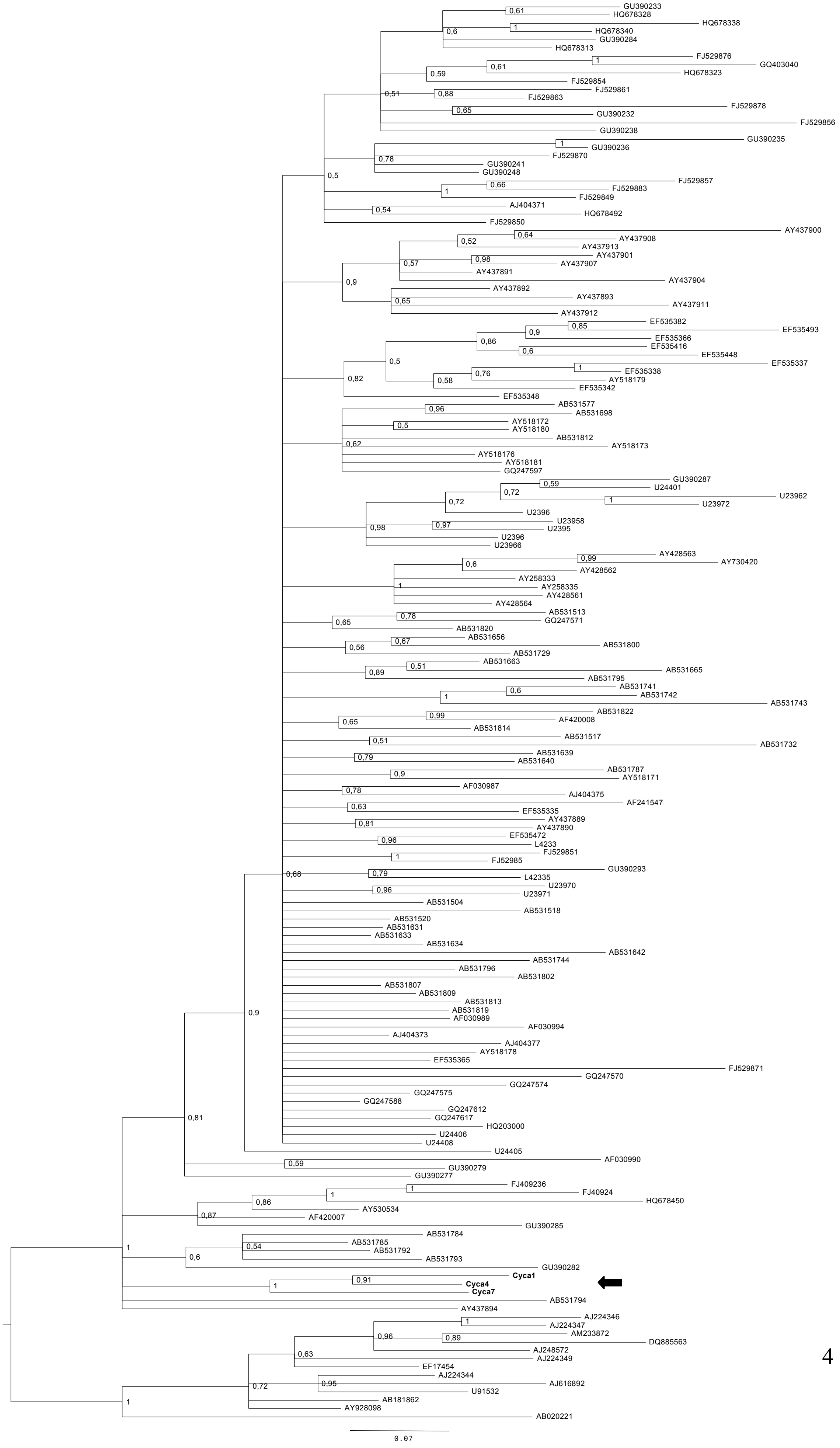

GU390233
0,61
HQ678328
HQ678338
0,6
1
HQ678340
GU390284
HQ678313
FJ529876
1
GQ403040
0,61
0,59
HQ678323
FJ529854
0,51
0,88
FJ529861
FJ529863
FJ529878
0,65
GU390232
FJ529856
GU390238
GU390235
1
GU390236
0,78
FJ529870
GU390241
GU390248
0,5
FJ529857
0,66
1
FJ529883
FJ529849
AJ404371
0,54
HQ678492
FJ529850
AY437900
0,64
AY437908
0,52
AY437913
AY437901
0,98
0,57
AY437907
AY437891
0,9
AY437904
AY437892
AY437893
0,65
AY437911
AY437912
EF535382
0,85
0,9
EF535493
0,86
EF535366
0,6
EF535416
EF535448
0,5
EF535337
1
0,76
EF535338
0,82
0,58
AY518179
EF535342
EF535348
AB531577
0,96
AB531698
AY518172
0,5
AY518180
AB531812
0,62
AY518173
AY518176
AY518181
GQ247597
GU390287
0,59
0,72
U24401
U23962
1
0,72
U23972
U2396
U23958
0,97
0,98
U2395
U2396
U23966
AY428563
0,99
0,6
AY730420
AY428562
AY258333
1
AY258335
AY428561
AY428564
AB531513
0,78
GQ247571
0,65
AB531820
AB531656
0,67
0,56
AB531800
AB531729
AB531663
0,51
0,89
AB531665
AB531795
AB531741
0,6
1
AB531742
AB531743
AB531822
0,99
0,65
AF420008
AB531814
AB531517
0,51
AB531732
AB531639
0,79
AB531640
AB531787
0,9
AY518171
AF030987
0,78
AJ404375
AF241547
0,63
EF535335
AY437889
0,81
AY437890
EF535472
0,96
L4233
FJ529851
1
FJ52985
GU390293
0,68
0,79
L42335
U23970
0,96
U23971
AB531504
AB531518
AB531520
AB531631
AB531633
AB531634
AB531642
AB531744
AB531796
AB531802
AB531807
AB531809
AB531813
0,9
AB531819
AF030989
AF030994
AJ404373
AJ404377
AY518178
EF535365
FJ529871
GQ247570
GQ247574
GQ247575
GQ247588
GQ247612
0,81
GQ247617
HQ203000
U24406
U24408
U24405
AF030990
0,59
GU390279
GU390277
FJ409236
1
1
FJ40924
0,86
HQ678450
AY530534
0,87
AF420007
GU390285
AB531784
1
AB531785
0,54
AB531792
0,6
AB531793
GU390282
Cyca1
0,91
Cyca4
1
Cyca7
AB531794
AY437894
AJ224346
1
AJ224347
0,96
AM233872
0,89
DQ885563
AJ248572
0,63
AJ224349
EF17454
AJ224344
0,95
AJ616892
0,72
U91532
1
AB181862
AY928098
AB020221
0.07
